# Protective Effects of Kaempferitrin on Advanced Glycation End Products Induce Mesangial Cell Apoptosis and Oxidative Stress

**DOI:** 10.3390/ijms19113334

**Published:** 2018-10-26

**Authors:** Wenxian Jiang, Rongshen Wang, Di Liu, Min Zuo, Chunzhen Zhao, Tianliang Zhang, Wanzhong Li

**Affiliations:** 1 School of Pharmacy, Weifang Medical University, Weifang 261053, China; jiangwenxian123@163.com (W.J.); wangrs199012@126.com (R.W.); ld0928928@163.com (D.L.); ZuoMin1026@163.com (M.Z.); zhaochunzhen@wfmc.edu.cn (C.Z.); 2 Experimental Center for Medical Research, Weifang Medical University, Weifang 261053, China; zhangtl@wfmc.edu.cn

**Keywords:** kaempferitrin, mesangial cells, advanced glycation end products, oxidative stress, apoptosis, signal pathway

## Abstract

Advanced glycation end products (AGEs) and the receptor for AGEs (RAGE) both play important roles in diabetic nephropathy (DN). Previous studies have identified glomerular mesangial cells (GMCs) injury as a key early risk factor in the development of DN. Kaempferitrin (KM) is a potent antioxidant with hypoglycemic action. Although KM is known to protect against AGE-induced damage in GMCs, the effects and the mechanisms by which they occur are poorly understood. In this study, cultured rat GMCs were exposed to AGE-induced oxidative stress (OS) to model DN in vitro. Reactive oxygen species (ROS) was analyzed by 2′,7′-dichlorofluorescin diacetate (DCFH-DA). Superoxide dismutase (SOD) and malondialdehyde (MDA) were studied using commercial kits. Mitochondrial membrane potential (Δψm) was measured by rhodamine 123. Hoechst 33258 and annexin V and propidium iodide (PI) double staining were performed to observe the apoptosis states in GMCs, whereas apoptosis and protective mechanism in AGE-induced GMCs were investigated by Western blot. The data revealed that KM effectively increased SOD activity, decreased MDA levels, suppressed ROS generation, and protected against OS in AGE-induced GMCs. Treatment with KM also inhibited the expression of collagen IV and transforming growth factor-β1 (TGF-β1), improved mitochondrial membrane potential recovery, and suppressed the mitochondrial/cytochrome c-mediated apoptosis pathway through the expression of anti-apoptotic factors in GMCs in vitro. These findings suggest that KM may be a new potential agent in the treatment of DN in future.

## 1. Introduction

Diabetes mellitus (DM) is a chronic metabolic disorder associated with long-term damage and failure of various organs [1]. Diabetic nephropathy (DN) is a serious and common chronic microvascular complication of DM [2], that is the main cause of mortality in patients with diabetes, as well as a major contributor to the high prevalence of end-stage renal disease (ESRD) worldwide [3]. The global prevalence of DM is growing rapidly, especially in developing countries, and the incidence rates of both DN and ESRD continue to increase [4]. Hyperglycemia is accompanied by an accelerated rate of formation of advanced glycation end products (AGEs). AGEs, which are derived from reducing the non-enzymatical reaction of sugars with amino groups of protein, play an important role in the pathogenesis of diabetic complications [5]. Despite recent progress, the underlying molecular mechanisms behind DM-induced renal damage are still poorly understood.

AGEs and RAGE both play a role in the pathogenesis of DN [6], triggering signaling cascade events and causing damage through oxidative stress (OS) [7]. OS is the result of excessive production of reactive oxygen species (ROS) [8], which can induce mitochondrial dysfunction, decrease adenosine triphosphate (ATP) production, and lead to the development of DN [9]. The transforming growth factor beta (TGF-β) family proteins are heavily involved in the progression of OS by regulating the expression levels of associated proteins and antioxidant enzymes [10]. AGEs accumulate in the glomerular basement membrane and in mesangial cells [11]. GMCs are key contributors to glomerulosclerotic and renal lesions in DM [12]. AGEs can influence cell function and induce OS and apoptosis both by interacting with receptors and by reducing basement membrane deformability, degradation, and matrix cell interaction [13]. However, the mechanisms behind AGE-induced GMCs injury, and the protective agents involved in the reduction of renal damage, have not been thoroughly explored.

Flavonoids are widely distributed in numerous flowering and non-flowering plants and are known as a potential source for new drugs due to their chemical diversities and low side-effect rates and toxicities [14]. Flavonoids are potent antioxidants, free radical scavengers [15] with anti-aging [16], neuroprotective [17], hypoglycemic actions [18,19], and anti-inflammatory properties [20]. Kaempferitrin (kaempherol-3, 7-bisrhamnoside; KM) is a naturally occurring flavonoid glycoside from many plants [21] that possesses antimicrobial activity [22], antitumor effect [23], antioxidative [24], and hypoglycemic activities [25,26]. It is thought that the antioxidative properties of flavonoids are potentially due to the ability of enzymes to obstruct OS [27]. Although they play a key role in the protection against apoptosis and OS, the effects of KM on AGE-induced injury of GMCs have not been characterized.

The use of AGE-induced renal cells as a model to study the cellular and molecular mechanisms of DN is widespread [28,29]. It is thought that AGEs may trigger OS, thereby accelerating GMCs apoptosis. However, the link between OS and apoptosis remains unclear. Therefore, our study aims to investigate the effects of KM on apoptosis and OS in AGE-stimulated mesangial cell and the underlying mechanism in vitro.

## 2. Results

### 2.1. KM Increases the Viability of AGE-Treated GMCs

**Figure 1:**
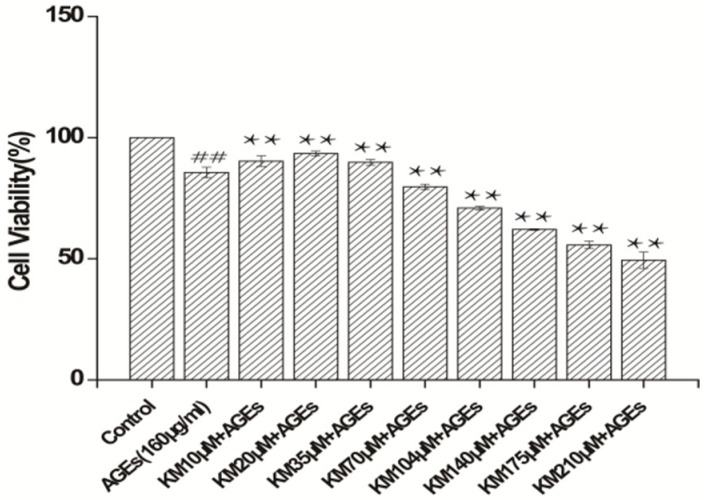
Kaempferitrin (KM) increases the cell viability of advanced glycation end (AGE)-treated glomerular mesangial cells (GMCs). GMCs were pre-treated with different concentrations of KM and then stimulated with AGEs (160 μg/mL) for 24 h. The results are presented as mean ± standard errors of the means (SEM). ** *p* < 0.01, vs. AGEs group; ^##^ *p* < 0.01, vs. control group.

To determine the effect that KM has on the viability of AGE-treated GMCs, cells were treated with different concentrations of KM (10, 20, 35, 70, 104, 140, 175, and 210 μM) or AGEs (160 μg/mL) for 24 h. The cell viability of GMCs treated with AGEs alone decreased as compared to that of the control group, (Figure 1; *p* < 0.01). Strikingly, the addition of even low concentrations of KM (10, 20, and 35 μM) to the AGE-treated GMCs rescued the AGE treatment phenotype, with the survival rate of cells treated with KM significantly increased as compared to that of the AGE-treated group (*p* < 0.01). KM at concentrations of 10, 20, and 35 μM were used for further experiments. These data show that KM, even at low concentrations, has a protective effect against AGE-induced decreases in GMC viability.

### 2.2. KM Treatment Protects against AGE-Induced GMC Injury

**Figure 2:**
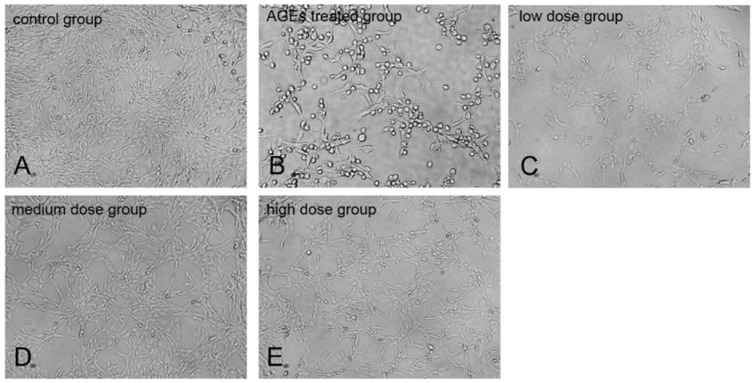
KM treatment protects against AGE-induced GMC injury. Microphotographs of the recovery effect of KM on AGE-induced growth of (**A**) control group GMCs, (**B**) cells treated with AGEs (160 μg/mL) for 24 h, and (**C**–**E**) cells pre-treated with 10, 20, or 35 μM KM and then stimulated with AGEs (160 μg/mL) for 24 h. The photographs were taken directly from culture plates with an inverted fluorescence microscope (magnification 100×).

Cells were pre-treated with 10, 20, or 35 μM KM for 24 h and then incubated with AGEs for 24 h. KM treatment ameliorated AGE-induced cell shrinkage and reduced the number of apoptotic vacuoles (Figure 2). The medium dose group (20 μM) recovery was more obvious after cell injury. These data indicate that KM treatment protects against AGE-induced injury of GMCs.

### 2.3. KM Treatment Reduced the Production of ROS in AGE-Treated GMCs

**Figure 3:**
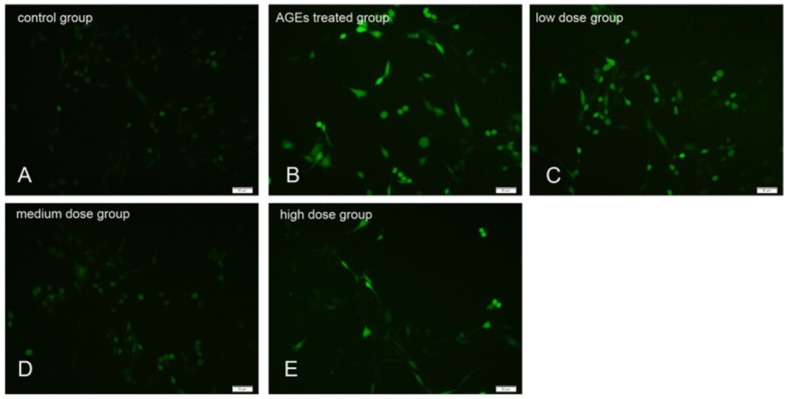
KM treatment reduces the production of ROS in AGE-treated GMCs. Detection of intracellular ROS using DCFH-DA in (**A**) control group cells, (**B**) cells treated with AGEs (160 μg/mL) for 24 h, and (**C**–**E**) cells pre-treated with 10, 20, or 35 μM KM and then stimulated with AGEs (160 μg/mL) for 24 h. The photographs were taken directly from culture plates with an inverted fluorescence microscope (magnification 50×).

To determine the effects of KM treatment on the levels of ROS, AGE-treated GMCs were exposed to 2′,7′-dichlorofluorescin diacetate (DCFH-DA), a green fluorescent dye. Treatment with AGEs resulted in an increase in green fluorescence compared to the untreated control group cells (Figure 3A,B), indicating that ROS production increased. Treatment of GMCs with low, medium, and high concentrations of KM (10, 20, and 35 μM, respectively) abated the green fluorescence (Figure 3C–E), indicating that ROS levels were reduced in these cells. These data show that KM treatment results in a decrease in ROS production in AGE-treated GMCs.

### 2.4. KM Treatment Improves Mitochondrial Membrane Potential Recovery in AGE-Treated GMCs

**Figure 4:**
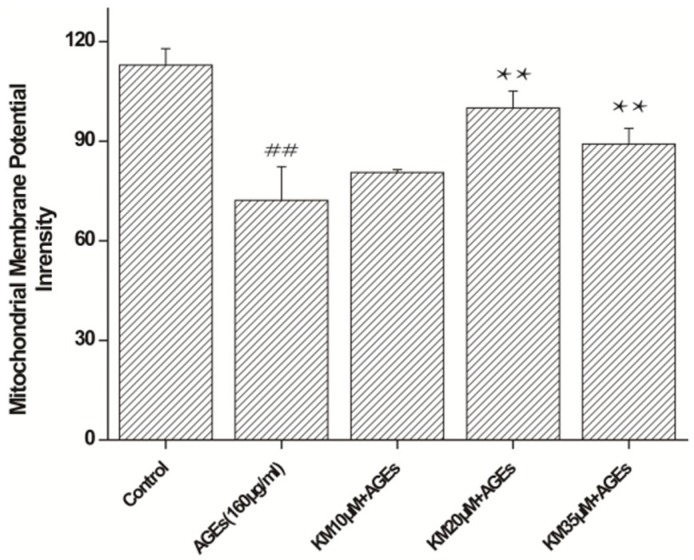
KM treatment improves mitochondrial membrane potential recovery in AGE-treated GMCs. Detection of the changes in mitochondrial membrane potential (∆Ψm) using rhodamine 123. GMCs were pre-treated with 10, 20, or 35 μM KM and then stimulated with AGEs (160 μg/mL) for 24 h. The results are presented as mean ± SEM. ** *p* < 0.01, vs. AGE group; ^##^ *p* < 0.01, vs. control group.

To determine whether KM affects AGE-induced apoptosis in GMCs through the mitochondrial apoptotic pathway, changes in the mitochondrial membrane potential (∆Ψm) were measured. Treatment of GMCs with AGEs resulted in a reduction in the mitochondrial membrane potential from 112.9 ± 5.0 to 72.2 ± 10.1 (Figure 4; *p* < 0.01). When AGE-treated cells were exposed to low, medium, or high concentrations of KM, the mitochondrial membrane potential showed recovery from the AGE control cells, and the membrane potential of the medium concentration KM group was 99.9 ± 5.1 (*p* < 0.01). These data show that KM treatment recovers the mitochondrial membrane potential of AGE-treated GMCs, indicating that KM rescues the apoptotic phenotype in these cells. 

### 2.5. KM Treatment Abates the Morphological Changes Induced by Apoptosis and Reduces the Rate of Apoptosis in AGE-Treated GMCs

**Figure 5:**
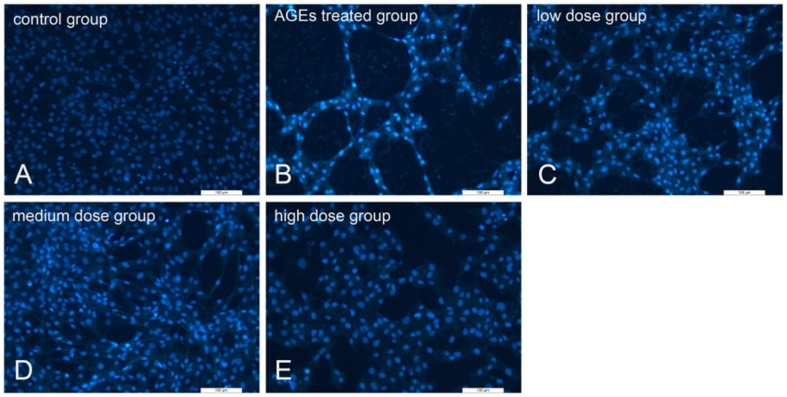
KM treatment prevents apoptosis-related morphological changes in the nuclei of AGE-treated GMCs. Detection of apoptotic morphology using Hoechst 33258 stain in (**A**) control group cells (**B**) cells treated with AGEs (160 μg/mL) for 24 h, and (**C**–**E**) cells pre-treated with 10, 20, or 35 μM KM and then stimulated with AGEs (160 μg/mL) for 24 h. The photographs were taken directly from culture plates with an inverted fluorescence microscope (magnification 100×).

To detect the effects of KM on the morphology of nuclei in AGE-treated GMCs, Hoechst 33258 staining was used. The nuclei of the untreated control group showed a uniform blue fluorescence, and the color was lightly distributed (Figure 5A). After the addition of AGEs, the staining of the nuclei was dense and fragmented, granular fluorescence was observed, indicating an increase in pyknosis (Figure 5B). KM treatment resulted in nuclear staining that was mostly normal, with only an occasional small part remaining densely stained (Figure 5C–E).

**Figure 6:**
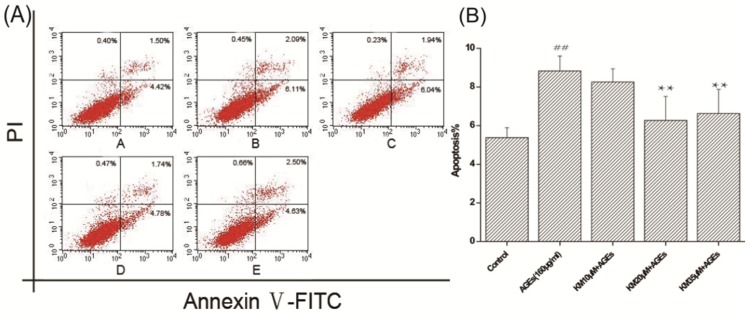
KM treatment reduces the rate of apoptosis in AGE-treated GMCs. Detection of apoptosis rate with annexin V and PI. Cells were stained with annexin V and PI after being treated with AGEs (160 μg/mL) and KM (10, 20, and 35 μM). (**A**) Percentages of apoptotic cells were determined by flow cytometry. FITC, Fluorescein Isothiocyanate. (**B**) The results were presented as mean ± SEM. ** *p* < 0.01, vs. AGEs group; ^##^ *p* < 0.01, vs. control group.

To determine how KM treatment affects the apoptotic rate of AGE-treated GMCs, annexin V and PI double staining was used. Figure 6 shows that there were a greater number of GMCs in early apoptosis and late apoptosis and that the apoptosis rate increased upon the addition of AGEs. Treatment with low, medium, or high concentrations of KM resulted in a decrease in the apoptotic rate of AGE-treated GMCs, with the middle dose group having the best results. These data indicate that KM treatment prevents apoptosis-associated morphological changes in the nuclei of and the rate of apoptosis in AGE-treated GMCs.

### 2.6. KM Modulates the Expression of Both Pro- and Anti-Apoptotic Proteins in GMCs Treated with AGEs

**Figure 7:**
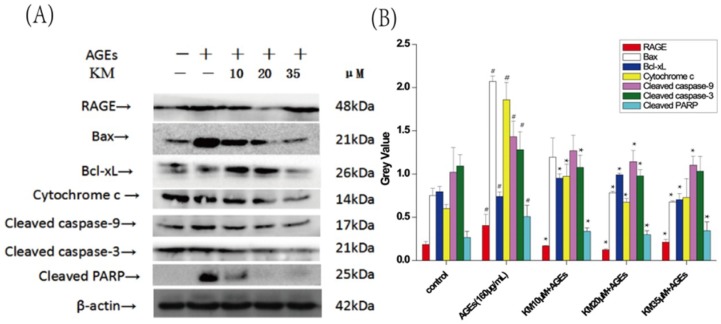
KM modulates the expression of both pro- and anti-apoptotic proteins in GMCs treated with AGEs. Effects of KM on AGE-induced apoptosis-related protein. GMCs were treated with AGEs (160 μg/mL) and KM (10, 20, or 35 μM). (**A**) The protein levels of RAGE, Bax, Bcl-xL and Cytochrome c, as well as the cleavage of caspases-9, -3 and poly-(ADP-ribose) polymerase (PARP), were examined by Western blot. + means adding reagents, − means not joining (**B**) The results were presented as mean ± SEM. * *p* < 0.05, vs. AGE group; ^#^ *p* < 0.05, vs. control group.

To determine the effects of KM on the expression of pro- and anti-apoptotic proteins in AGE-treated GMCs, Western blot analysis was used. Upon treatment with AGEs, the level of Bcl-xL was significantly reduced, whereas the level of Bax was significantly increased (Figure 7). Strikingly, treatment with KM resulted in the decrease in the level of Bax. The involvement of cytochrome c and caspase activation was also investigated. The levels of cleaved caspases-9 and -3 increased upon treatment with AGEs, but decreased when KM was added. Furthermore, AGEs induced the expression of RAGE and increased the cleaved form of poly-(adenosine diphosphate-ribose) polymerase (PARP), whereas treatment with KM reduced its expression. These results indicate that KM regulates AGE-induced apoptosis via a mitochondrial apoptotic pathway.

### 2.7. KM Treatment Enhances Antioxidation and Reduces the Proliferation of the Extracellular Matrix in AGE-Treated GMCs

To determine the degree of the antioxidant activity of KM possesses in AGE-treated cells, analyses using superoxide dismutase (SOD) and malondialdehyde (MDA) kits were combined. SOD activity was reduced in GMCs treated with AGEs (*p* < 0.05) but was increased in response to treatment with KM, particularly by the medium KM dose (Table 1). This finding indicates that KM restores the activity of cells to remove free radicals. Moreover, AGEs increased the MDA content in GMCs (*p* < 0.05), which corresponds to cellular damage of cells. By contrast, KM treatment decreased the MDA content, with the medium dose treatment having the most significant results.

**Figure 8:**
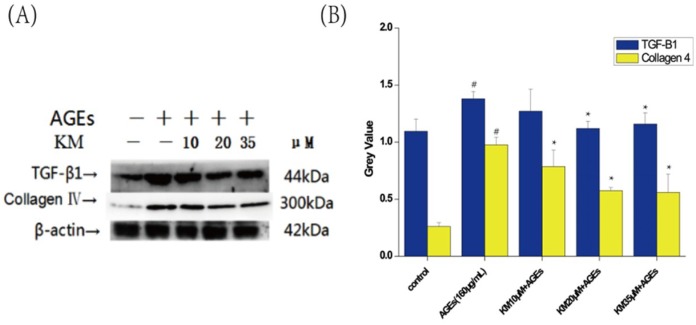
KM treatment reduces the proliferation of the ECM in AGE-treated GMCs. GMCs were treated with AGEs (160 μg/mL) and KM (10, 20 or 35 μM). (**A**) The protein levels of TGF-β1 and Collagen IV were examined by Western blot. + means adding reagents, − means not joining (**B**) The results were presented as mean ± SEM. * *p* < 0.05, vs. AGEs group; ^#^ *p* < 0.05, vs. control group.

To determine the effect of KM treatment on AGE-induced changes in the extracellular matrix (ECM) of GMCs, Western blot analyses were used. Expression levels of TGF-β1 and Collagen IV were increased in AGE-treated GMCs compared to untreated control cells. Treatment with low, medium, or high doses of KM resulted in the decrease in the expression levels of TGF-β1 and Collagen IV, consequently reducing the expansion of the ECM (Figure 8).

## 3. Discussion

The early stages of DN are characterized by renal hypertrophy, glomerular hyperfiltration, and microalbuminuria [30]. These changes are related to the subsequent development of glomerular morphological abnormalities and the diagnosis of DN. There is a growing interest in the beneficial roles of KM in the prevention of DM and its complications. As such, we investigated the protective effects of KM against AGE-induced apoptosis and OS in GMCs in vitro.

Extracellular AGEs bind to RAGE, which induces the generation of intracellular ROS and stimulates the production and release of cytokines [31]. TGF-β expression is induced by the increase in glucose levels that occur in response to OS induction and AGEs production [32]. TGF-β1 augments the deposition of ECM proteins, such as several types of collagens, fibronectin, and laminin at the glomerular level. Therefore, AGEs subsequently induce hypertrophy and fibrosis in GMCs via ROS [33,34]. AGEs play a key role in the development of DM and DM-related complications, as well as promote the deterioration of renal function [35]. Therefore, targeting AGEs could be a suitable therapeutic approach for the prevention of DN.

OS is not only a consequence of AGEs–RAGE activation but also a potent inducer of RAGE expression [36]. Thus, antioxidants should be considered as a treatment strategy for AGEs–RAGE-related diseases such as DN. SOD activity and MDA content are two major indices in the oxidative balance system [37]. KM possesses phenolic hydroxyl groups and natural antioxidative properties, both of which increase SOD activity and decrease MDA content in AGE-induced GMCs. KM also inhibits the expression of TGF-β1 and collagen IV proteins, which may abate the ROS-related damage to GMCs that occurs in DN.

Recent studies have shown that ROS are potent inducers of mitochondrial apoptotic signaling pathways. The maintenance of the mitochondrial membrane potential (Δψm) is crucial for mitochondrial function. The collapse of ΔΨm and depletion of ATP are the direct consequences of ROS accumulation in mitochondria, and these events precede the release of cytochrome c and activation of the caspase protease cascade [38]. We have found that AGEs induce a significant depletion in the ΔΨm of GMCs, a result that is in agreement with those reported in previous studies [39]. ROS-mediated ΔΨm depletion and mitochondrial dysfunction are essential for the induction of AGE-induced apoptosis in GMCs. Upon depletion of ΔΨm, there is an AGE-induced activation of caspase-3, which increases the expression of cytochrome c and its release from the mitochondria.

The release of cytochrome c from mitochondria has been shown to be an almost universal phenomenon during celluar apoptosis. The mitochondria-initiated intrinsic pathway requires the release of cytochrome c and promotes the caspase-activating apoptosome, a complex that induces the activation of caspase-9 and initiates the apoptotic caspase cascades [40]. Caspase-3 is regarded as the apoptosis promoter [41]. Caspases have been shown to be activated during apoptosis in many cells and play key roles in both the initiation and execution of apoptosis [42]. Mitochondrial apoptosis is regulated mainly by the Bcl-2 family of cell death regulatory molecules, which include the pro-apoptotic Bax and the anti-apoptotic Bcl-2 [43]. 

**Figure 9:**
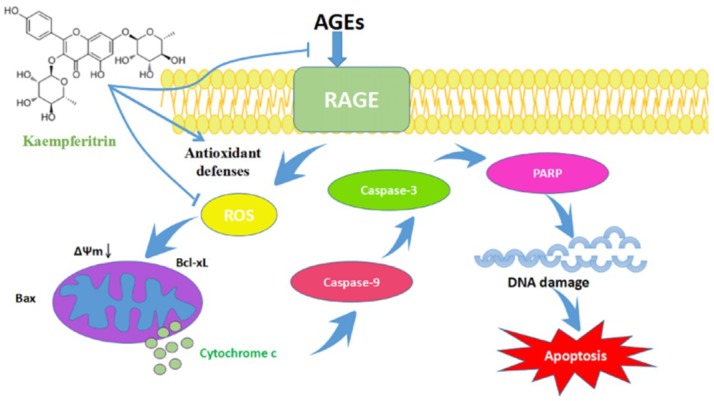
Schematic representation of the biological activities of KM and its effects on the AGE-induced mitochondrial/cytochrome c-mediated apoptosis pathway in GMCs. The thick blue arrows represent the order of action, the thin blue arrows represent the effects of drug intervention, the black arrow represents the decrease in mitochondrial membrane potential, the light green dots represent the release of cytochrome c in mitochondria, and the different color is for differentiate between different parts and proteins in the cell.

Hyperglycemia and OS can trigger an increase in Bax levels, which activates the apoptosis-signaling pathway, including the cleavage of both caspase-3 and caspase-9 [44]. AGEs-induced ROS production in the mitochondria results in cytochrome c release through the regulation of Bax and Bcl-xL [45,46,47]. Thus, cytochrome c could activate caspase-9 and caspase-3 and stimulate the proteolytic cleavage of PARP, resulting in morphological and membrane potential changes and subsequent mitochondrial and nuclear DNA damage [48,49]. In this study, we found that KM reduces the production of ROS and inhibits the AGE-induced apoptosis of GMCs (Figure 9).

In this study, we explored the effects of KM treatment on AGE-induced injury of GMCs in vitro. The effects of KM on glomerular injury using animal models of DN would greatly enhance the understanding of the protective effects of KM, as well as demonstrate its feasibility as a treatment for DN. Moreover, the in vitro experimental conditions used in our studies did not fully reflect in vivo conditions. Further studies are needed to clarify whether KM treatment could prevent glomerular damage in human DN by blocking the AGEs–RAGE axis and the mitochondrial-mediated apoptotic signaling pathway. Our research contributes to the growing evidence indicating its potential usefulness in treating or managing other diabetic complications. The data reported in this study will facilitate future studies that investigate the in vivo bioavailability and pharmacokinetics of KM, as well as determine the full extent of its protective effects and any toxic side effects.

## 4. Materials and Methods

### 4.1. Reagents

KM (lot NO., 17020911, purity > 98%) was purchased from Manster Biotechnology Co., Ltd. (Cheng du, China). Dulbecco’s modified Eagle’s medium (DMEM) and fetal bovine serum (FBS) were purchased from KeyGen Biotechnology Co., Ltd. (Nanjing, China). Bovine serum albumin (BSA), D-glucose and 3-(4,5-Dimethylthiazol-2-yl)-2,5-diphenyltetrazolium bromide (MTT) were obtained from Sigma-Aldrich (St. Louis, MO, USA). Penicillin, streptomycin, rhodamine 123, hoechst 33258, reactive oxygen species assay kit and Bicinchoninicacid (BCA) protein concentration determination kit were purchased from Solarbio Technology Co., Ltd. (Beijing, China). Anti-rat RAGE (ab65965), Bax (ab32503), Bcl-xL (ab32370), Cytochrome C (ab133504), Cleaved-caspase-3 (ab13847), Cleaved-caspase-9 (ab25758), Cleaved-PARP (ab32138), TGF-β1 (ab92486), Collagen IV (ab6586) and β-actin (ab8227) were purchased from Abcam Biotechnology Co., Ltd. (Shanghai, China). The annexin V and PI apoptosis kits were purchased from Invitrogen Thermo Fisher Scientific (Carlsbad, CA, USA). The AGEs Enzyme-linked Immuno Sorbent Assay (ELISA) kit was obtained from R&D Systems (Minneapolis, MN, USA). The superoxide dismutase (SOD) kit and Malondialdehyde (MDA) kit were purchased from Jiancheng Bioengineering Institute (Nanjing, China).

### 4.2. AGEs Preparation

AGEs-BSA conjugation was prepared by incubating 5 g bovine serum albumin with 9 g D-glucose in 10 mL of phosphate buffer solution (PBS) (0.2M, pH 7.4) for 8 weeks at 37 °C and the free glucose was dialyzed in 0.01 M PBS (pH 7.4) for 96 h [50]. The identification of AGEs was determined at excitation 370 nm and emission 440 nm in a Gemini EM fluorescence microplate reader (Molecular Devices, Sunnyvale, CA, USA). The content of AGEs was determined using an AGEs ELISA kit [51].

### 4.3. Cell Culture

The rat glomerular mesangial cell (GMC) line was purchased from KeyGen Biotechnology (Nanjing, China) and cultured in Dulbecco’s modified Eagle’s medium supplemented with 10% fetal bovine serum, 100 U/ml penicillin and 100 mg/ml streptomycin at 37 °C in 5% CO_2_. The medium was replaced every 48 h until the cells reached 80% confluency [52].

### 4.4. MTT Assay

GMCs were seeded in 96-well culture plates at 5 × 10^3^ cells/mL and then incubated for 24 h. Cells were then treated as follows: (1) Control medium, (2) AGEs (160 μg/mL), (3) different concentrations of KM. After another 24 h treatment, the culture solution was removed and 5 mg/mL of the MTT solution was added to each well for 4 h. The medium was then removed and 100 μL dimethyl sulfoxide (DMSO) was added to dissolve the formed formazan. The plates were detected at 490 nm using a microplate reader (Molecular Devices, Sunnyvale, CA, USA).

### 4.5. Cellular Morphology Analysis

GMCs were seeded in six-well culture plates at 10 × 10^4^ cells/mL and then incubated for 24 h. Cells were then treated as follows: (1) Control medium, (2) AGEs (160 μg/mL), (3) KM (10 μM) + AGEs (160 μg/mL), (4) KM (20 μM) + AGEs (160 μg/mL), and (5) KM (35 μM) + AGEs (160 μg/mL). After 24 h treatment, the morphological changes were observed under an inverted fluorescence microscope (Olympus, Tokyo, Japan).

### 4.6. Determination of SOD and MDA Content

The SOD and MDA antioxidant indices were measured using the corresponding kits. All procedures were performed in accordance with the manufacturer’s instructions. Briefly, GMCs were treated as before, then lyse cells by freeze-thaw cycles, cell supernatants were collected, and SOD and MDA levels were determined according to the manufacturer’s instructions.

### 4.7. Detection of Intracellular ROS Accumulation

Intracellular ROS accumulation was monitored using DCFH-DA, which is a specific fluorescent probe [53]. GMCs were seeded in six-well plates and treated as described in Section 4.5. The cells were collected and incubated with 20 μM DCFH-DA at 37 °C for 30 min in the dark, and then observed under an inverted fluorescence microscope (Olympus, Tokyo, Japan).

### 4.8. Assessment of Mitochondrial Membrane Potential

The mitochondrial membrane potential (∆Ψm) was determined according previously described methods [54]. Briefly, after treatment according to Section 4.5, GMCs were loaded with 10 μM rhodamine 123 and incubated at 37 °C for 30 min in the dark. Cells were then harvested, washed, and analyzed with the excitation wavelength at 488 nm and the emission wavelength at 525 nm by a microplate reader (Molecular Devices, Sunnyvale, CA, USA).

### 4.9. Hoechst 33258 Staining

Apoptotic morphology was observed using Hoechst 33258 staining [55]. GMCs were treated in groups according to Section 4.5, washed with pre-cooled PBS three times, and incubated in the dark for 10 min at 37 °C with 20 μM Hoechst 33258. The cells were observed under an inverted fluorescence microscope (Olympus, Tokyo, Japan).

### 4.10. Annexin V and PI Double Staining

The rate of apoptosis was determined by annexin V and PI double staining [56]. After the GMCs were treated in groups according to Section 4.5, the cells were collected and centrifuged at 1000 rpm for 5 min at 4 °C. Following two PBS washes, the cells were resuspended in 100 μL 1X binding buffer containing 5 μL PI and 5 μL FITC-annexin V for 15 min in the dark. Then, 400 μL 1X binding buffer was replenished and the samples were analyzed using flow cytometry (BD Biosciences, Franklin Lake, NV, USA). A total of 10,000 cells were examined per sample, and both early (PI negative and annexin V positive stained cells) and late (PI positive and annexin V positive stained cells) apoptotic cells were analyzed.

### 4.11. Subcellular Fractionation for the Detection of Cytochrome C Release

Subcellular fractionation was used to extract cytochrome c [57]. The cells were collected and washed with cold PBS. 500 μL of lysate was ground on a mortar and centrifuged at 1000× *g* for 10 min at 4 °C. The supernatant was collected and centrifuged at 12,000× *g* for 30 min at 4 °C. The lower layer precipitate was mitochondria, and it was collected and further lysed before analysis by Western blotting.

### 4.12. Western Blotting

The cells were harvested and lysed with radio immunoprecipitation assay (RIPA) buffer (10 mM Tris/HCl, pH 7.5, 150 mM NaCl, 1% Nonidet P-40) containing 1 mM phenylmethanesulfonyl fluoride (PMSF), 1 mM ethylene diamine tetraacetic acid (EDTA), 1 μg/ml pepstatin and 1 μg/mL aprotinin. The protein content was measured using a BCA protein kit. For each sample, 100 μg of the lysate were loaded into the sodium dodecyl sulfate polyacrylamide gel electrophoresis (SDS-PAGE) gel and transferred to a polyvinylidene fluoride (PVDF) membrane. The membrane was incubated with 5% of skim milk powder dissolved in Tris-buffered saline with Tween 20 (TBST) for 1 h. The membrane was then incubated with anti-RAGE, Bax, Bcl-xL, Cytochrome c, Cleaved-caspase-3, Cleaved-caspase-9, PARP, TGF-β1, or Collagen IV (1:1000), overnight at 4 °C. The membrane was then washed three times with TBST and incubated with horseradish peroxidase-conjugated anti-rabbit immunoglobulin G (IgG) (1:5000) for 2 h at room temperature. The signals were detected with the chemiluminescence system. Immunoreactive bands were quantitatively analyzed using Alpha View software (Fluor Chem Q).

### 4.13. Statistical Analysis

All data were presented as mean ± SEM. The statistical analyses were performed using SPSS 19.0 software (SPSS Inc., Chicago, IL, USA). Analysis of variance (ANOVA) was used to assess the significant differences between multiple groups. Statistical significance was expressed as *p* < 0.05.

## 5. Conclusions

The results of this study revealed that KM increases SOD activity, decreases MDA levels, reduces ROS production, and inhibits the expression of collagen IV and TGF-β1. Moreover, these data show that KM suppresses the mitochondrial/cytochrome c-mediated apoptosis pathway by inducing the expression of anti-apoptotic proteins in GMCs in vitro. These findings suggest that KM may be a viable agent for the prevention and treatment of DN.

## Figures and Tables

**Table 1:** Effects of KM treatment on AGE-induced MDA and SOD content in GMCs.

Groups	SOD (U/mL)	MDA (n mol/mL)
Control	1.29 ± 0.067	0.51 ± 0.006
AGEs group	0.65 ± 0.006 ^#^	0.89 ± 0.009 ^#^
KM low-dose group	0.81 ± 0.007 *	0.78 ± 0.020 *
KM medium-dose group	0.93 ± 0.021 *	0.58 ± 0.012 *
KM high-dose group	0.76 ± 0.005 *	0.69 ± 0.007 *

^#^*p* < 0.05, vs. control group; * *p* < 0.05, vs. AGEs group.
